# Lack of association of MRI determined subclinical cardiovascular disease with dizziness and vertigo in a cross-sectional population-based study

**DOI:** 10.1371/journal.pone.0184858

**Published:** 2017-09-14

**Authors:** Roberto Lorbeer, Holger Hetterich, Ralf Strobl, Anina Schafnitzel, Hannah Patscheider, Andreas Schindler, Katharina Müller-Peltzer, Wieland Sommer, Annette Peters, Christa Meisinger, Margit Heier, Wolfgang Rathmann, Fabian Bamberg, Eva Grill

**Affiliations:** 1 Department of Radiology, University Hospital, LMU Munich, Munich, Germany; 2 Institute of Medical Information Processing, Biometrics and Epidemiology, LMU Munich, Munich, Germany; 3 Institute of Epidemiology II, Helmholtz Zentrum München, Neuherberg, Germany; 4 German Center for Diabetes Research (DZD e.V.), Neuherberg, Germany; 5 German Center for Cardiovascular Disease Research (DZHK e.V.), Munich, Germany; 6 Chair of Epidemiology, LMU Munich, UNIKA-T Augsburg, Augsburg, Germany; 7 KORA Myocardial Infarction Registry, Central Hospital of Augsburg, Augsburg, Germany; 8 Department of Biometrics and Epidemiology, German Diabetes Center, Duesseldorf, Germany; 9 Department of Diagnostic and Interventional Radiology, Eberhard Karls University Tübingen, Tübingen, Germany; 10 German Center for Vertigo and Balance Disorders, LMU Munich, Munich, Germany; 11 Munich Center of Health Sciences (MC-Health), LMU Munich, Munich, Germany; Worcester Polytechnic Institute, UNITED STATES

## Abstract

**Objective:**

We investigated the association between subclinical cardiovascular diseases assessed by MRI examination and symptoms of dizziness and vertigo in participants of a population-based sample.

**Methods:**

Data from 400 participants (169 women) aged from 39 to 73 of a cross-sectional MRI sub-study of the “Kooperative Gesundheitsforschung in der Region Augsburg” (KORA) FF4 study from the south of Germany was used. MRI determined subclinical cardiovascular diseases include left and right ventricular structure and function as well as the presence of carotid plaque and carotid wall thickness. Cerebrum diseases include white matter lesions (WML) and cerebral microbleeds (CMB). The main outcomes of dizziness and vertigo were assessed by standardized interview. Logistic regression models were applied and adjusted odds ratios (OR) with 95% confidence intervals (CI) were provided.

**Results:**

Lifetime and 12-month prevalence of dizziness and vertigo were 30% (95%CI 26% to 35%) and 21% (95%CI 17% to 26%) respectively in this sample. On multivariable analysis, cardiac and carotid measurements were not associated with dizziness and vertigo excluding orthostatic vertigo (20%, 95CI 16% to 24%). Only in male participants, there was a significant association between WML and the presence of dizziness and vertigo (OR = 2.95, 95%CI 1.08 to 8.07). There was no significant association of CMB with dizziness and vertigo.

However, CMB and WML were tending to associate with a higher risk of dizziness and vertigo in the whole sample (CMB: OR = 1.48, 95%CI 0.70; 3.15; WML: OR = 1.71, 95%CI 0.80 to 3.67;), in persons with prediabetes and diabetes (WML: OR = 2.71, 95%CI 0.89 to 8.23) and in men with normal glucose metabolism (CMB: OR = 2.60, 95%CI 0.56 to 12.0; WML: OR = 3.08, 95%CI 0.58 to 16.5).

**Conclusions:**

In this sample of participants without manifest cardiovascular diseases, subclinical left and right ventricular function and carotid structure were consistently not associated with dizziness and vertigo. Subclinical cerebrum measurements, however, tend to increase the risk for dizziness and vertigo, especially in men and in persons with prediabetes or diabetes.

## Introduction

Dizziness and vertigo are among the most common complaints in the general population leading to a medical consultation [1]. Symptoms increase with age [2] and are associated with disability and reduced quality of life [3]. Peripheral vestibular disorders are frequent causes for dizziness and vertigo with benign paroxysmal positioning vertigo as the most frequent form of peripheral vestibular disorders with a lifetime prevalence of 2% in the general population [4]. Other, less-frequent peripheral forms of vestibular disorders include Menière’s disease and vestibular neuritis. Central vestibular forms of vertigo include cerebrovascular diseases, brain stem and cerebellar lesions, infections, and vestibular migraine [5]. In aged adults, the ageing of vestibular and proprioceptive systems and, most notably, medication are potential risk factors for dizziness and vertigo [6].

Besides this, several studies have demonstrated an association of dizziness and vertigo with abnormal heart rhythm, congestive heart failure, heart valve failure and stroke [5–7]. Although the association seems straightforward, these conditions are rather rare [5] and the independency of other risk factors is often not clear. In a population-based study, angina pectoris, myocardial infarction and stroke were associated with vestibular vertigo only when adjusted for age and sex, but not in further multivariable adjusted analysis, in which hypertension appeared as an independent cardiovascular risk factor for vestibular vertigo [1].

In most of the studies investigating causes of dizziness and vertigo, cardiovascular diseases had been measured by standardized medical interviews asking for self-reported diagnosed prevalence [1, 7]. Magnetic resonance imaging (MRI) provides an alternative method to measure a broad range of cardiovascular phenotypes with a higher validity and with continuous subclinical characteristics. Therefore, the aim of the present analysis was to investigate the association between subclinical cardiovascular disease assessed by MRI examination and symptoms of dizziness and vertigo in healthy subjects of a community-based sample.

## Methods

### Study sample

Data of the study project “Kooperative Gesundheitsforschung in der Region Augsburg” (KORA) FF4 from the south of Germany was used for the present analysis. Of all 4261 participants of the KORA S4 baseline study, 2279 residents of the city of Augsburg and two surrounding counties also participated in the 14-year follow-up FF4 study conducted between 2013 and 2014 [8]. In a FF4 MRI sub-study, a number of 400 FF4 participants (169 women) aged 39 to 73 years without validated stroke, myocardial infarction, arterial vessel occlusion or any contraindication for MRI examinations reported previously [9] could be examined by MRI. Sample size differed between cardiovascular exposure groups of left ventricular function (N = 381), right ventricular function (N = 365), carotid plaque (N = 264) and cerebrum abnormalities (N = 386) due to insufficient image quality and missing imaging data.

The investigations were carried out in accordance with the Declaration of Helsinki, including written informed consent of all participants. All study methods were approved by the ethics committee of the Bavarian Chamber of Physicians, Munich (S4: EC No. 99186 and for genetic epidemiological questions 05004, F4 and FF4: EC No. 06068). The MRI examination protocol was further approved by the ethics committee of the Ludwig-Maximilian-University Hospital, Munich.

### MRI examination and cardiovascular measurements

All examinations were performed at a 3 Tesla Magnetom Skyra (Siemens AG, Healthcare Sector, Erlangen Germany) using an 18 channel body coil in combination with the table-mounted spine matrix coil. Subject position was supine. All subjects underwent an imaging protocol that comprised following sequences: 4-chamber view steady state free precession (SSFP), Short-axis stack SSFP (cardiac imaging); axial blackblood T1 weighted fat saturated (T1w fs ax) (carotid plaque); three dimensional T2 weighted fluid attenuated inversion recovery (T2w FLAIR 3D), and axial susceptibility weighted imaging (SWI ax) (brain).

#### Left ventricular function

The cine-SSFP sequences were evaluated semi-automatically using commercially available software (cvi42, Circle Cardiovascular Imaging, Calgary, Canada). Following automatic contour detection of the left ventricular endocardial and epicardial border, all borders were corrected manually, if necessary. End-diastolic and end-systolic phase were identified. Established left ventricular volumetric data were derived according to current guidelines [10] and included end-diastolic volume and end-systolic volume with calculated stroke volume (end-diastolic volume minus end-systolic volume) and ejection fraction ((stroke volume/end-diastolic volume)*100) as well as end-systolic and end-diastolic myocardial mass.

#### Right ventricular function

Right ventricular function parameters were derived by manual segmentation of the right ventricular endocardial border on axial cine-SSFP sequences using dedicated software (cvi42, Circle Cardiovascular Imaging, Calgary, Canada). Parameters included end-diastolic volume and end-systolic volume with calculated stroke volume and ejection fraction.

#### Assessment of carotid plaque

Presence of plaque, measures of plaque burden and gross composition of carotid plaque were determined on black-blood T1w fs ax in both carotid arteries according to the modified American Heart Association (AHA) classification system [11]. Presence of plaque was defined as eccentric plaque (i.e. wall thickening) with or without calcification (AHA lesion type III–VII). Boundaries of the vessel lumen and the vessel wall were analysed using a commercially available semiautomatic dedicated software tool (CASCADE, University of Washington, Seattle, US) [12]. If necessary, manual corrections were performed. Based on these, values for wall area and lumen area, as well as maximum wall thickness were derived. For analysis of plaque burden irrespective of vessel size the normalized wall-index was calculated by division of wall area by the entire vessel area.

#### White matter lesions and microbleeds

White matter lesions (WML) were graded on T2w FLAIR 3D images according to the age-related white matter changes scale (ARWMC) [13] in five brain areas in the left and right hemisphere, respectively. In each of the areas the ARWMC score can take values from 0 to 3. Presence of WML was defined as an ARWMC score>1 in any of the analysed areas. In addition, for sensitivity analysis, WML degree variables were calculated. First, ARWMC score was categorized as 0: ARWMC score = 0; 1: ARWMC score = 1; 2: ARWMC score = 2; 3: ARWMC score = 3 (as the maximum score in any of the ten analysed areas). Second, total ARWMC score summed scores of all areas, thereby ranging from 0 to 30 and total ARWMC score was further categorized as: 0: total ARWMC score = 0; 1: total ARWMC score = 1|2; 2: total ARWMC score = 3|4; 3: total ARWMC score = 5|6; 4: total ARWMC score>6.

Cerebral microbleeds (CMB) were defined as small, foci of signal loss (≥2 mm) on SWI ax and counted in the lobar, deep, or infratentorial left or right hemisphere. Symmetric signal loss in the globus pallidum, most likely calcification, flow void artifact of the pial blood vessels, and intracerebral lesions with a hemorrhagic component were excluded. Besides investigating total CMB, participants were also divided into groups of people with microbleeds in lobar brain regions only, and people with microbleeds in deep or infratentorial regions.

### Measurements of dizziness and vertigo

Prevalence of dizziness and vertigo was measured by standardised face-to-face interview using questions from the balance section of the National Health and Nutrition Examination Survey Questionnaire for life-time prevalence: “Have you ever had vertigo or dizziness or difficulty with balance?” and 12 months prevalence: ‘‘During the last 12 months, have you had vertigo or dizziness or difficulty with balance?”. We used a combination of dizziness and vertigo as outcome since study participants quite often are not able to distinguish between dizziness and vertigo symptoms and further neuro-otological diagnostic was not available. For main analyses, 12 months prevalence of dizziness and vertigo excluding orthostatic vertigo was used. Individuals were defined as suffering from orthostatic vertigo if vertigo was only triggered by standing up too fast.

### Co-variables

Health related co-variables were assessed by standardized interview, basic health examinations, laboratory analyses or medication record. Smoking status differentiated between never-smoker, ex-smoker and current smoker. Body mass index (BMI) was calculated as weight divided by squared height (kg/m^2^) and waist circumference was measured in cm to the closest 0.1 cm at the smallest position between the lower rib and the upper margin of the iliac crest. Body surface area (BSA) was calculated according to the Du Bois formula [14]: (BSA = 0.007184×(height in cm)^0.725^×(weight in kg)^0.425^.

Systolic and diastolic blood pressure (BP) were measured three times at the right arm of seated participants after a five-minute resting period and three-minute resting periods between reading. The mean of the second and third BP measurement was used for the present analyses. An oscillometric digital BP monitor (HEM-705CP, Omron Corporation, Tokyo, Japan) was used and one of two cuff sizes was applied. Hypertensive participants had an increased systolic BP (≥140 mmHg), an increased diastolic BP (≥90 mmHg) or used antihypertensive medication under awareness of having hypertension.

Diabetes was defined as a 2-hour plasma glucose concentration measured by oral glucose tolerance test (OGTT) ≥200 mg/dl and/or a fasting glucose level ≥125 mg/dl. Prediabetes was characterized as a 2-hour serum glucose concentration measured by OGTT ranging between 140 and 199 mg/dL and as a fasting glucose level between 110 and 124 mg/dL. Subjects without diabetes or prediabetes were described as subjects with normal glucose metabolism. Laboratory measurements including glucose and HbA1c as well as total cholesterol, high- and low-density lipoprotein cholesterol were described elsewhere [15].

### Statistical analyses

Baseline characteristics of subjects with and without lifetime prevalence of dizziness and vertigo were displayed as absolute numbers and percentages or median and interquartile range according to categorical or continuous data types. Characteristic differences were evaluated by χ^2^-test or by Wilcoxon rank-sum test.

Logistic regression models were used to assess the associations between subclinical cardiovascular measurements and lifetime prevalence of dizziness and vertigo and 12-month prevalence of dizziness and vertigo excluding orthostatic vertigo. Continuous independent variables were divided by their standard deviation (SD) to demonstrate a SD increment in risk factors. Adjusted odds ratios (OR) with 95% confidence intervals (CI) were provided. Associations of cardiovascular measurements with dizziness and vertigo were adjusted for age, sex, BMI, hypertension and diabetes mellitus to exclude confounding bias. Left and right ventricular structure and function were additionally indexed by BSA. Linearity assumption of logistic regression was verified by testing multivariable regression spline models. In further analyses, statistical models were additionally adjusted for sampling weights considering differences between the study sample (n = 400) and the entire KORA cohort (n = 2279, median age = 60 years, 48% men, 15% diabetics) without changed findings. In sensitivity analyses, logistic regression models were realized separately for women and men as well as for different diabetic status. A p-value <0.05 was used to determine statistical significance. Data were analyzed by statistical software of Stata 14.1 (Stata Corporation, College Station, TX, U.S.A.).

## Results

Lifetime prevalence of dizziness and vertigo was 30% (95%CI 26% to 35%) in this sample. Study participants with lifetime dizziness and vertigo were slightly older (median age = 58 years), had a higher proportion of women (50%) and never-smokers (45%) compared to participants without lifetime dizziness and vertigo (56 years, p = 0.372; 39% women, p = 0.030; 33% never-smoker, p = 0.005; Table 1). BMI, systolic and diastolic BP as well as HbA1c levels did not differ between the two groups. More baseline characteristics of the study sample are presented in Table 1.

**Table 1:** Baseline characteristics of the study sample according to lifetime prevalence of dizziness and vertigo.

	With dizziness and vertigo	Without dizziness and vertigo	p-value
	N = 121	N = 279	
Age (years)	58 (50–64)	56 (48–64)	0.372
Men	60 (49.6%)	171 (61.3%)	0.030
Smoking status			0.005
Never-smoker	54 (44.6%)	92 (33.0%)	
Ex-smoker	54 (44.6%)	120 (43.0%)	
Current smoker	13 (10.7%)	67 (24.0%)	
BMI (kg/m^2^)	27.1 (24.5–30.8)	27.5 (24.8–30.9)	0.927
BSA (m^2^)	1.9 (1.8–2.1)	2.0 (1.8–2.1)	0.045
Waist circumference (cm)	98 (89–107)	100 (90–107)	0.553
Systolic BP (mmHg)	120 (109–131)	121 (108–131)	0.861
Diastolic BP (mmHg)	75 (69–81)	75 (68–81)	0.932
Hypertension	39 (32.2%)	97 (34.8%)	0.623
Diabetic status			0.305
Normal glucose metabolism	79 (65.3%)	164 (58.8%)	
Prediabetes	25 (20.7%)	78 (28.0%)	
Diabetes	17 (14.1%)	37 (13.3%)	
HbA1c (%)	5.4 (5.2–5.6)	5.4 (5.3–5.7)	0.360
Glucose (mg/dl)	98 (91–109)	99 (93–110)	0.630
HDL-C (mg/dl)	62 (49–75)	59 (48–72)	0.172
LDL-C (mg/dl)	139 (120–162)	137 (114–160)	0.620
Total cholesterol (mg/dl)	220 (193–248)	214 (190–239)	0.253

Data are given as number (percentage) or median (25^th^ and 75^th^ percentile).

p-values from χ^2^ test or Wilcoxon rank-sum test

BMI, body mass index; BSA, body surface area; BP, blood pressure; HbA1c, hemoglobin A1c; HDL-C, high-density-lipoprotein cholesterol; LDL-C, low-density-lipoprotein cholesterol.

### Cardiovascular MRI parameters

The cardiac functions stroke volume and ejection fraction of left and right ventricle were similar between groups with and without lifetime dizziness and vertigo. Right ventricle end-diastolic volume and left ventricle myocardial masses were non-significantly lower in participants with dizziness and vertigo compared to the other group Table 2. Presence rate of right and left carotid artery plaque was higher in participants without dizziness and vertigo (25% vs. 14%, p = 0.036), whereas normalized wall index and mean wall thickness were similar between both groups. In the analysis for cerebrum characteristics, the presence of WML and CMB were more prevalent in participants with dizziness and vertigo (14%, 15%, respectively) compared to participants without dizziness and vertigo (11%, 12%; Table 2) but without statistical significance.

**Table 2:** Cardiovascular MRI characteristics of the study sample according to lifetime prevalence of dizziness and vertigo.

MRI parameter	With dizziness and vertigo	Without dizziness and vertigo	p-value
*Left ventricle*	*N = 112*	*N = 269*	
End-diastolic volume (ml)	128 (103–152)	124 (108–149)	0.702
End-systolic volume (ml)	38 (29–51)	36 (29–49)	0.710
Stroke volume (ml)	87 (75–103)	87 (74–101)	0.963
Ejection Fraction (%)	71 (64–74)	70 (64–75)	0.756
Myocardial mass, diastolic (g)	134 (115–164)	143 (119–161)	0.287
Myocardial mass, systolic (g)	137 (112–163)	144 (118–167)	0.192
*Right ventricle*	*N = 108*	*N = 257*	
End-diastolic volume (ml)	159 (129–186)	167 (139–193)	0.115
End-systolic volume (ml)	76 (56–94)	77 (61–95)	0.387
Stroke volume (ml)	85 (70–97)	87 (73–100)	0.311
Ejection Fraction (%)	53 (48–58)	53 (48–58)	0.773
*Carotid artery (right and left)*	*N = 89*	*N = 175*	
Presence of plaque (AHA lesion type III-VII) (%)	12 (13.5%)	43 (24.6%)	0.036
Normalized wall index	0.43 (0.40–0.45)	0.42 (0.40–0.45)	0.689
Mean wall thickness (mm)	0.73 (0.68–0.80)	0.73 (0.69–0.79)	0.853
*Cerebrum*	*N = 117*	*N = 269*	
Presence of white matter lesions (%)	16 (13.7%)	30 (11.2%)	0.482
Presence of cerebral microbleeds (%)	18 (15.4%)	31 (11.7%)	0.314

Data are given as number (percentage) or median (25^th^ and 75^th^ percentile).

p-values from χ^2^ test or Wilcoxon rank-sum test

### Association of cardiovascular MRI parameters with dizziness and vertigo

**Fig 1:**
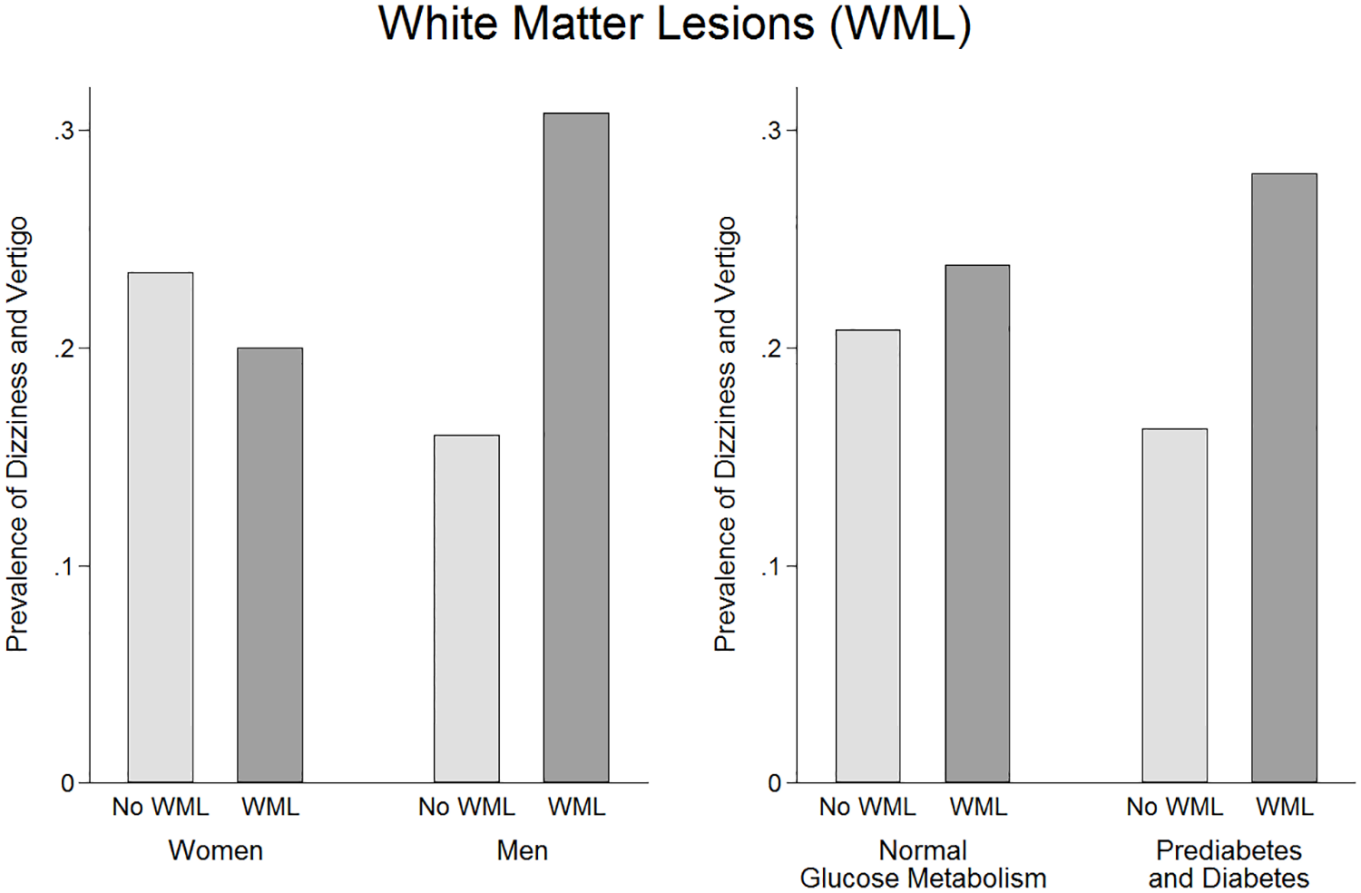
Prevalence of 12-month dizziness and vertigo according to groups with and without white matter lesions. In women and men (Panel A) and in persons with normal glucose metabolism and in prediabetic or diabetic persons (Panel B).

**Fig 2:**
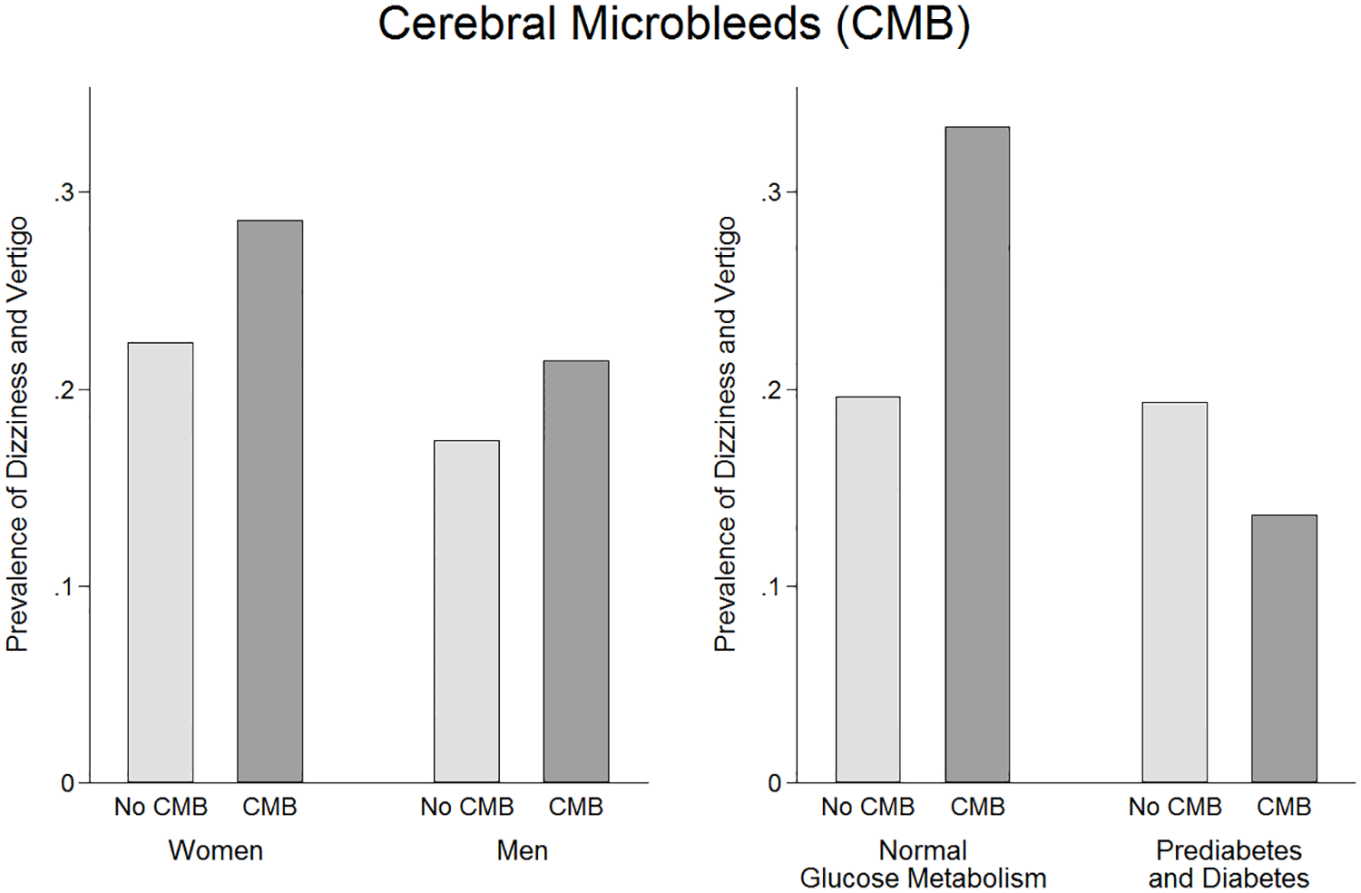
Prevalence of 12-month dizziness and vertigo according to groups with and without cerebral microbleeds. In women and men (Panel A) and in persons with normal glucose metabolism and in prediabetic or diabetic persons (Panel B).

Multivariable adjusted logistic regression models did not reveal any association between cardiovascular MRI phenotypes and dizziness and vertigo (Table 3). The highest non-significant ORs for lifetime dizziness and vertigo and 12-month dizziness and vertigo excluding orthostatic vertigo were observed for presence of WML (OR = 1.26, 95%CI 0.63 to 2.49; OR = 1.71, 95%CI 0.80 to 3.67; respectively) and CMB (OR = 1.40, 95%CI 0.72 to 2.70; OR = 1.48, 95%CI 0.70 to 3.15). In sensitivity analyses, the relation between WML and 12-month dizziness and vertigo revealed only significance in men (OR = 2.95, 95%CI 1.08 to 8.07), and showed higher non-significant effects in persons with prediabetes or diabetes (OR = 2.71, 95%CI 0.89 to 8.23) (Fig 1) and in men with normal glucose metabolism (OR = 3.08, 95%CI 0.58 to 16.5). Using WML degree variables, no significant associations between degree of WML and dizziness and vertigo were detected either in the whole sample (S1 Table) or in subgroups of sex and diabetes status (data not shown). The relation between CMB and 12-month dizziness and vertigo revealed to be non-significantly stronger in men (OR = 1.58, 95%CI 0.55 to 4.54) (Fig 2) and in men with normal glucose metabolism (OR = 2.60, 95%CI 0.56 to 12.0). Differentiating between lobar CMB and deep or infratentorial CMB did not change the non-significant results substantially (S2 Table).

**Table 3:** Association of MRI determined cardiovascular measurements with dizziness and vertigo.

MRI parameter*	Dizziness and vertigo			
	Lifetime prevalence		12-month prevalence	
	Odds Ratio (95% confidence interval)	p-value	Odds Ratio (95% confidence interval)	p-value
*Left ventricle*				
End-diastolic volume/BSA (ml/m^2^)	1.13 (0.89–1.44)	0.307	0.99 (0.75–1.32)	0.957
End-systolic volume/BSA (ml/m^2^)	1.14 (0.91–1.44)	0.249	1.12 (0.87–1.45)	0.372
Stroke volume/ BSA (ml/m^2^)	1.06 (0.84–1.35)	0.615	0.87 (0.65–1.16)	0.341
Ejection Fraction (%)	0.93 (0.74–1.17)	0.560	0.89 (0.69–1.15)	0.371
Myocardial mass, diastolic/BSA (g/m^2^)	1.18 (0.91–1.53)	0.221	1.10 (0.81–1.51)	0.536
Myocardial mass, systolic/BSA (g/m^2^)	1.11 (0.84–1.46)	0.482	1.07 (0.77–1.50)	0.688
*Right ventricle*				
End-diastolic volume/BSA (ml/m^2^)	0.94 (0.72–1.23)	0.666	0.86 (0.62–1.18)	0.343
End-systolic volume/BSA (ml/m^2^)	0.99 (0.77–1.28)	0.957	0.98 (0.72–1.32)	0.876
Stroke volume/ BSA (ml/m^2^)	0.94 (0.73–1.22)	0.635	0.80 (0.58–1.10)	0.166
Ejection Fraction (%)	0.99 (0.76–1.29)	0.944	0.89 (0.65–1.22)	0.462
*Carotid artery (right and left)*				
Presence of plaque (AHA lesion type III-VII) (%)	0.50 (0.24–1.02)	0.058	0.44 (0.17–1.12)	0.085
Normalized wall index	1.03 (0.79–1.35)	0.810	1.02 (0.75–1.39)	0.916
Mean wall thickness (mm)	1.07 (0.81–1.4)	0.645	1.07 (0.77–1.47)	0.691
*Cerebrum*				
Presence of WML (%)	1.26 (0.63–2.49)	0.512	1.71 (0.80–3.67)	0.169
Presence of CMB (%)	1.40 (0.72–2.70)	0.319	1.48 (0.70–3.15)	0.304

Data are from logistic regression, adjusted for age, sex, BMI, hypertension and diabetes mellitus.

* SD increment for continuous variables.

BSA, body surface area; WML, white matter lesions; CMB, cerebral microbleeds.

## Discussion

This first investigation of the role of several subclinical cardiovascular and cerebrovascular disease parameters for symptoms of dizziness and vertigo in a population-based sample revealed no association of MRI determined left and right ventricular function as well as carotid artery structure, WML and CMB with 12-month prevalence of dizziness and vertigo excluding exclusive orthostatic vertigo. However, the cerebrovascular parameters WML and CMB were slightly but not significantly increased in participants with dizziness and vertigo and especially in men and prediabetic and diabetic participants.

### Left and right ventricular heart function

The finding of no association of reduced left and right ventricular function with dizziness is in line with a population-based study of around 3000 participants aged 65 years and older that observed no association between self-reported cardiovascular disease and dizziness problems [7]. However, in the study above, self-reported abnormal heart rhythm was associated with increased risk of dizziness (OR = 1.9, 95%CI 1.2 to 2.8). Another study of around 5000 participants found an increased risk for vestibular vertigo in participants with self-reported coronary heart disease (OR = 2.1, 95%CI 1.1 to 3.8) and cardiovascular disease (OR = 2.0, 95%CI 1.1 to 3.6) including angina pectoris and myocardial infarction after adjustment for age and sex but without statistical significance after multivariable adjustment [1]. In our study, we excluded participants with overt cardiovascular disease to characterize subclinical cardiovascular disease status, and we used more sensitive MRI measurements of continuous heart function parameters.

Although cardiovascular diseases are observed and discussed as causes of dizziness and vertigo in the literature reviews of studies with patients revealed that they were overall relatively uncommon (e.g. cardiac arrhythmia 1.5%) [5, 6]. Heart valve disease and arrhythmia are mainly discussed as causes for the dizziness symptom of presyncope [16]. Another cause for presyncope seems to be orthostatic hypotension with BP drop on standing [16]. Orthostatic vertigo defined as vertigo after standing up too fast was excluded in our study to focus on non-orthostatic subclinical cardiovascular diseases.

### Cerebrovascular parameters

Cerebrovascular diseases have a higher potential to cause dizziness compared to cardiac dysfunction, although it is also low with 6% of all dizziness cases in which 2.9% of all cases were due to stroke and 2.6% due to transient ischemic attack (TIA) [5]. In a population-based setting of patients with dizziness and vertigo symptoms, the symptom of imbalance appeared as a predictor for stroke and TIA independently of age and sex [17]. Furthermore, dizziness of abrupt onset and multiple prodromal episodes of dizziness were predictive for stroke in patients with acute vestibular syndrome (AVS) [18].

Similarly to investigating cardiac function, we excluded participants with stroke as an overt event to characterize subclinical cerebrovascular disease status. A previous study demonstrated that already small strokes (lesions≤10 mm) can be present together with isolated AVS [19].

Imaging examinations including MRI and computed tomography are often applied to visualize brain abnormalities in dizzy patients [16]. Large WML were observed to be associated with different balance-related symptoms whereas the influence of small lesions is still unclear [16]. In our study, the presence of WML was defined as ARWMC ratings 2 (beginning confluence of lesions) or 3 (diffuse involvement of the entire region) and showed by trend a higher risk for 12-month prevalence of dizziness and vertigo. This finding supports the tendency of an association of cerebrovascular disease and dizziness and vertigo. Furthermore, the latter finding is supported by our result by trend of non-significantly increased dizziness and vertigo prevalence in participants with CMB.

A contribution of WML as verified by MRI to the occurrence of vertigo was also suggested by a study of patients with visual vertigo [20]. Another study found a higher frequency of severe WML in a patients group with unexplained dizziness compared to a group of explained causes of dizziness [21]. In addition, there is evidence that dizziness might be more indicative of WML than of silent brain infarcts [22]. The association between CMB and dizziness and vertigo is less discussed in the literature. However, CMB were reported to be associated with postural instability measured as the inability to balance on one leg for a reasonable time [23].

### Carotid artery structure

Data for a possible independent association of carotid artery structure including stenosis, carotid plaque and wall thickness with dizziness and vertigo are rare. Although the impact of carotid artery stenosis is described as being low and integrated in the general cardiovascular risk for dizziness [24, 25], some case reports emphasize dizziness as a leading symptom in patients with carotid artery occlusion [26, 27]. Carotid plaque, normalized wall index and mean carotid wall thickness seem to be of minor importance for predicting dizziness and vertigo in our study.

### Strengths and limitations

For this analysis, MRI was used to assess a broad range of cardiovascular phenotypes allowing well validated continuous measurements. Since this study was nested in the established KORA cohort project, standardized methods were also available for measuring dizziness and vertigo as well as the confounding variables BMI and hypertension. The population-based design is well-suited to investigate subclinical ranges of cardiovascular disease as risk factors for dizziness and vertigo. Nevertheless, some limitations should be considered. The study sample of N = 400 is reasonable but might be too small to detect especially small effects of subclinical cerebrovascular parameter differences on dizziness and vertigo. Power analysis revealed a detectable dizziness and vertigo proportion difference of 19% points (corresponding OR = 2.6) between control group (N = 354; dizziness and vertigo proportion = 19%) and the group with WML (N = 46; 38%) with a power of 80%. The MRI sub-study was designed as a nested case-control study to investigate primarily differences in subclinical cardiovascular disease between persons with diabetes, prediabetes and controls. Thus, the representativeness of the study is not given and results could be biased by selection. However, additional analyses using weights accounting for differences between MRI sub-study and the whole cohort did not reveal different results. Furthermore, no effect modification of diabetic status was found for the association between subclinical cardiovascular diseases and dizziness and vertigo except borderline significant associations of WML and CMB with dizziness and vertigo in persons with prediabetes or diabetes and in men with normal glucose metabolism, respectively.

### Conclusion

In this large sample of participants without manifest cardiovascular diseases examined in parallel by MRI and symptom assessment of dizziness and vertigo, subclinical left and right ventricular function and carotid structure were consistently not associated with dizziness and vertigo. Subclinical cerebrum measurements, however, tend to increase the risk for dizziness and vertigo, especially in men and in persons with prediabetes or diabetes. This slight tendency of association needs to be validated in larger study samples. The findings support the minor role of cardiovascular diseases as risk factors for dizziness and vertigo.

## Supporting information

S1 Table Association of MRI determined *degree of white matter lesions* with dizziness and vertigo. Data are from logistic regression, adjusted for age, sex, BMI, hypertension and diabetes mellitus. *Reference: ARWMC score = 0; 1: ARWMC score = 1; 2: ARWMC score = 2; 3: ARWMC score = 3 (in any of the ten analyzed areas). **Reference: Total ARWMC score = 0; 1: total ARWMC score = 1|2; 2: total ARWMC score = 3|4; 3: total ARWMC score = 5|6; 4: total ARWMC score>6.
(DOCX)

S2 Table Association of MRI determined *cerebral microbleeds* with dizziness and vertigo. Data are from logistic regression, adjusted for age, sex, BMI, hypertension and diabetes mellitus.
(DOCX)
