# Supplementary material for: Lack of association of MRI determined subclinical cardiovascular disease with dizziness and vertigo in a cross-sectional population-based study
Source: PLoS One. 2017 Sep 14;12(9):e0184858. doi: 10.1371/journal.pone.0184858 (PMC5599022; doi:10.1371/journal.pone.0184858)
Supplement: S1 Table — Data are from logistic regression, adjusted for age, sex, BMI, hypertension and diabetes mellitus. *Reference: ARWMC score = 0; 1: ARWMC score = 1; 2: ARWMC score = 2; 3: ARWMC score = 3 (in any of the ten analyzed areas). **Reference: Total ARWMC score = 0; 1: total ARWMC score = 1|2; 2: total ARWMC score = 3|4; 3: total ARWMC score = 5|6; 4: total ARWMC score>6. (DOCX) [file pone.0184858.s001.docx]

**S1 Table.** Association of MRI determined *degree of white matter lesions* with dizziness and vertigo.

|  |  | Dizziness and Vertigo | | | | | | |  |
| --- | --- | --- | --- | --- | --- | --- | --- | --- | --- |
|  |  | Lifetime prevalence | | |  | 12-month prevalence | | |  |
| MRI parameter | N | Odds Ratio  (95% confidence interval ) | p-value |  | | | Odds Ratio  (95% confidence interval ) | p-value | |
| *White matter lesions (degree)* |  |  |  |  | | |  |  | |
| Definition 1* |  |  |  |  | | |  |  | |
| 0 | 139 | 1 |  |  | | | 1 |  | |
| 1 | 201 | 0.76 (0.45-1.27) | 0.291 |  | | | 0.71 (0.39-1.30) | 0.262 | |
| 2 | 43 | 1.05 (0.48-2.30) | 0.908 |  | | | 1.31 (0.54-3.17) | 0.556 | |
| 3 | 3 | 0.85 (0.07-10.6) | 0.898 |  | | | 1.97 (0.15-25.2) | 0.603 | |
| Definition 2** |  |  |  |  | | |  |  | |
| 0 | 139 | 1 |  |  | | | 1 |  | |
| 1 | 79 | 0.66 (0.34-1.26) | 0.204 |  | | | 0.51 (0.23-1.13) | 0.098 | |
| 2 | 54 | 1.02 (0.50-2.08) | 0.956 |  | | | 1.03 (0.45-2.34) | 0.941 | |
| 3 | 57 | 0.75 (0.36-1.57) | 0.445 |  | | | 0.83 (0.35-1.93) | 0.660 | |
| 4 | 57 | 0.89 (0.43-1.86) | 0.765 |  | | 1.12 (0.48-2.58) | | 0.798 | |

Data are from logistic regression, adjusted for age, sex, BMI, hypertension and diabetes mellitus.

*Reference: ARWMC score=0; 1: ARWMC score=1; 2: ARWMC score=2; 3: ARWMC score=3 (in any of the ten analyzed areas).

**Reference: Total ARWMC score=0; 1: total ARWMC score=1|2; 2: total ARWMC score=3|4; 3: total ARWMC score=5|6; 4: total ARWMC score>6.
