# Supplementary material for: Lack of association of MRI determined subclinical cardiovascular disease with dizziness and vertigo in a cross-sectional population-based study
Source: PLoS One. 2017 Sep 14;12(9):e0184858. doi: 10.1371/journal.pone.0184858 (PMC5599022; doi:10.1371/journal.pone.0184858)
Supplement: S2 Table — Data are from logistic regression, adjusted for age, sex, BMI, hypertension and diabetes mellitus. (DOCX) [file pone.0184858.s002.docx]

**S2 Table.** Association of MRI determined *cerebral microbleeds* with dizziness and vertigo.

|  |  | Dizziness and Vertigo | | | | | |
| --- | --- | --- | --- | --- | --- | --- | --- |
|  |  | | Lifetime prevalence | |  | 12-month prevalence | |
| MRI parameter | N | | Odds Ratio  (95% confidence interval ) | p-value |  | Odds Ratio  (95% confidence interval ) | p-value |
| *Cerebral microbleeds* |  | |  |  |  |  |  |
| None | 334 | | 1 |  |  | 1 |  |
| Strictly lobar | 32 | | 1.52 (0.68-3.37) | 0.304 |  | 1.62 (0.65-4.02) | 0.301 |
| Deep or infratentorial | 17 | | 1.20 (0.42-3.46) | 0.734 |  | 1.28 (0.39-4.23) | 0.688 |

Data are from logistic regression, adjusted for age, sex, BMI, hypertension and diabetes mellitus.
